# Antimicrobial Therapy for Pneumonia in the Emergency Department: The Impact of Clinical Pharmacists on Appropriateness

**DOI:** 10.5811/westjem.2017.5.33901

**Published:** 2017-07-10

**Authors:** Brett A. Faine, Nicholas Mohr, Jenna Dietrich, Laura Meadow, Kari K. Harland, Elizabeth Chrischilles

**Affiliations:** * University of Iowa, College of Pharmacy, Iowa City, Iowa; † University of Iowa, College of Public Health, Iowa City, Iowa; ‡ University of Iowa, Department of Emergency Medicine, Iowa City, Iowa; § University of Iowa, Department of Epidemiology, Iowa City, Iowa; ¶ University of Iowa Hospitals and Clinics, Department of Emergency Medicine, Iowa City, Iowa; || University of Iowa Hospitals and Clinics, Department of Pharmacy, Iowa City, Iowa; # University of Iowa, Hospitals and Clinics, Department of Anesthesia, Iowa City, Iowa; ** Carilion Roanoke Memorial Hospital, Department of Critical Care Pharmacy, Roanoke, Virginia

## Abstract

**Introduction:**

Pneumonia impacts over four million people annually and is the leading cause of infectious disease-related hospitalization and mortality in the United States. Appropriate empiric antimicrobial therapy decreases hospital length of stay and improves mortality. The objective of our study was to test the hypothesis that the presence of an emergency medicine (EM) clinical pharmacist improves the timing and appropriateness of empiric antimicrobial therapy for community-acquired pneumonia (CAP) and healthcare-associated pneumonia (HCAP).

**Methods:**

This was a retrospective observational cohort study of all emergency department (ED) patients presenting to a Midwest 60,000-visit academic ED from July 1, 2008, to March 1, 2016, who presented to the ED with pneumonia and received antimicrobial therapy. The treatment group consisted of patients who presented during the hours an EM pharmacist was present in the ED (Monday-Friday, 0900–1800). The control group included patients presenting during the hours when an EM clinical pharmacist was not physically present in the ED (Monday–Friday, 1800–0900, Saturday/Sunday 0000–2400 day). We defined appropriate empiric antimicrobial therapy using the Infectious Diseases Society of America consensus guidelines on the management of CAP, and management of HCAP.

**Results:**

A total of 406 patients were included in the final analysis (103 treatment patients and 303 control patients). During the hours the EM pharmacist was present, patients were significantly more likely to receive appropriate empiric antimicrobial therapy (58.3% vs. 38.3%; p<0.001). Regardless of pneumonia type, patients seen while an EM pharmacist was present were significantly more likely to receive appropriate antimicrobial therapy (CAP, 77.7% vs. 52.9% p=0.008, HCAP, 47.7% vs. 28.8%, p=0.005). There were no significant differences in clinical outcomes.

**Conclusion:**

The presence of an EM clinical pharmacist significantly increases the likelihood of appropriate empiric antimicrobial therapy for patients presenting to the ED with pneumonia.

## INTRODUCTION

Over four million Americans are diagnosed with pneumonia annually, and it is the leading cause of infectious disease-related hospitalization and mortality in the United States. 1–3 Empiric antimicrobial therapy is often initiated in the emergency department (ED) and pneumonia remains one of the most common infections requiring antimicrobial therapy.^4^,^5^ Further, the treatment of pneumonia is complex, with changing antimicrobial susceptibilities, changing definitions, and changing time-to-treatment targets making uniform appropriate treatment challenging.^6^,^7^ Appropriate antimicrobial therapy for community-acquired pneumonia (CAP) and healthcare-associated pneumonia (HCAP) has been shown to decrease hospital length of stay and mortality. 8, 9 Infectious Diseases Society of America (IDSA) guidelines provide guidance for the treatment of CAP and HCAP and recommend identifying patients with risk factors for multi-drug resistant (MDR) pathogens to select empiric therapy.^6^,^7^ Unfortunately, in a busy ED setting, emergency medicine (EM) providers are left with the difficult task of differentiating patients at risk for MDR pathogens to select appropriate antimicrobial therapy. EM clinical pharmacists play an important role on the healthcare team and have been shown to impact antimicrobial prescribing for various infectious conditions. 10–14 A clinical pharmacist in the ED has a unique focus on pharmacotherapy prescribing, allowing them to assess the patient for multi-drug resistant (MDR) pathogen risk factors and guide empiric antimicrobial therapy.

The primary objective of our study was to test the hypothesis that the presence of an EM clinical pharmacist improves the appropriateness of empiric antimicrobial therapy for CAP and HCAP. Secondary objectives were to assess whether the presence of an EM clinical pharmacist improves timing antimicrobial therapy and if appropriate antimicrobial therapy shortened hospital length of stay (LOS), decreased repeat hospital visits for pneumonia, and reduced in-hospital mortality.

## METHODS

### Design

This study was a retrospective observational cohort study conducted in the ED of an academic medical center with an annual ED census of 60,000 patient visits between July 1, 2008, and March 1, 2016.

### Participants and setting

We included all patients 18 years and older diagnosed with pneumonia who received antimicrobial therapy in the ED and were admitted to the hospital. Patients were identified by *International Classification of Diseases, Ninth Revision (ICD-9)* discharge diagnosis codes for pneumonia. During the data abstraction process, the diagnosis of pneumonia was confirmed by the ED provider’s documentation in the electronic medical record (EMR). Patients were excluded if they had a diagnosis of cystic fibrosis, did not receive antimicrobial therapy in the ED, or had incomplete documentation in their medical records.

Population Health Research Capsule

What do we already know about this issue?

Appropriate antimicrobial therapy for pneumonia decreases hospital length of stay and mortality. Emergency medicine (EM) pharmacists have been shown to impact antimicrobial prescribing.

What was the research question?

Does the presence of an EM pharmacist improve appropriateness of empiric antimicrobial therapy for pneumonia?

What was the major finding of the study?

EM pharmacist presence increases the likelihood of appropriate empiric antimicrobial therapy for patients with pneumonia.

How does this improve population health?

EM pharmacists play an important role in the healthcare team and can have a positive impact on medication appropriateness for patients presenting to the emergency department.

The treatment group consisted of patients who received antimicrobial therapy during the hours an EM clinical pharmacist was present in the ED (before October 2015: Monday–Friday, 0900–1800; starting October 2015: Monday–Saturday, 0900–1900). The control group included patients who received antimicrobial therapy during the hours when an EM clinical pharmacist was not physically present in the ED.

All variables were defined *a priori* and recorded in an EMR as part of clinical care. Variables collected from the patient’s EMR included age, height, weight, gender, date and time of presentation, past medical history, serum creatinine, white blood cell count, lactate, risk factors for MDR-resistant pathogens, initial antimicrobial therapy administered in the ED, time to antimicrobial therapy, mechanical ventilation in the ED, admitting service (general ward vs. intensive care unit), hospital LOS, in-hospital mortality and 30-day repeat hospital visits for pneumonia. Clinical variables were abstracted from the EMR by a trained data abstractor (LM) blinded to the study hypothesis. After data abstraction, 10% of charts were randomly selected for review by a second independent pharmacist (JD) to validate data accuracy and abstraction techniques.

### Definitions

We defined appropriate empiric antimicrobial therapy using the IDSA consensus guidelines on the management of CAP and management of HCAP.^6^,^7^ Appropriate vancomycin dosing was defined as 15–20 mg/kg in accordance with guideline recommendations. 15 An independent clinical pharmacist unaware of patient group allocation (treatment vs. control group) determined appropriateness of antimicrobial therapy based on IDSA guidelines. Patients were defined as receiving guideline-concordant therapy if they met all criteria in the guidelines (e.g. ceftriaxone plus azithromycin = appropriate for CAP, ceftriaxone monotherapy = inappropriate for CAP). We defined risk factors for MDR pathogens as hospitalization for two days or more in the preceding 90 days, residence in a long-term care facility or nursing home, chronic hemodialysis, home infusion therapy (including antibiotics), chronic home wound care, and immunosuppressive disease/therapy. 7 The definition of immunosuppressive disease/therapy included the following: patients taking corticosteroids (at least 5 mg per day of prednisone or an equivalent drug) or immunomodulating agent (e.g infliximab, adalimumab, etanercept, etc.), documentation of human immunodeficiency virus, received either a solid organ transplant or bone marrow transplant, or were receiving treatment with chemotherapy or radiation.

### Outcomes

The primary outcome was the proportion of patients who received appropriate empiric antimicrobial therapy for CAP and HCAP. Secondary outcomes included time to antimicrobial therapy, appropriate vancomycin dosing, hospital LOS, 30-day repeat visits for pneumonia and in-hospital mortality. We also measured the effect of antimicrobial selection in the ED and whether the same empiric antimicrobial therapy was continued upon admission to the hospital.

### Analysis

We calculated that a sample size of 90 patients per group would have 80% power (α = 0.05) to detect an absolute difference of 20% in patients who receive appropriate antimicrobial therapy, assuming that antimicrobial appropriateness was 26% in the control group based on previous reports in the literature.^8^,^10^ To examine differences in patient characteristics and outcomes by the absence/presence of the EM pharmacists, we reported Pearson chi-square and percent differences (95% confidence interval [CI]) for categorical variables. Differences in continuous variables (e.g., age), were examined using mean differences and 95% CIs (parametric variables) or Wilcoxon sum test (non-parametric variables). We used multivariable logistic regression analysis to estimate the effect of clinical pharmacist presence on 30-day repeat visits among survivors, controlling for potentially confounding covariates (age, antimicrobial appropriateness, CAP, HCAP). We prespecified variables included in the model based on *a priori* knowledge and defined a statistical threshold for inclusion of p<0.20. All tests were two-tailed and a p-value <0.05 was considered statistically significant. We conducted all analyses using SAS^®^ software (version 9.3, SAS system for Microsoft, SAS institute Inc., Cary, NC, USA). The institutional review board approved the study protocol. The design and results reporting were completed in accordance with the Strengthening the Reporting of Observational Studies in Epidemiology (STROBE) statement.^16^

## RESULTS

We included 406 patients in the final analysis (103 treatment group and 303 control group). There were no statistically significant differences in demographic variables (Table 1). Hospitalization for two or more days in the prior 90 days (n=131, 52%) and immunosuppressive disease/therapy (n=112, 45%) were the most prevalent risk factors for HCAP (Table 2).

**Figure 1:**
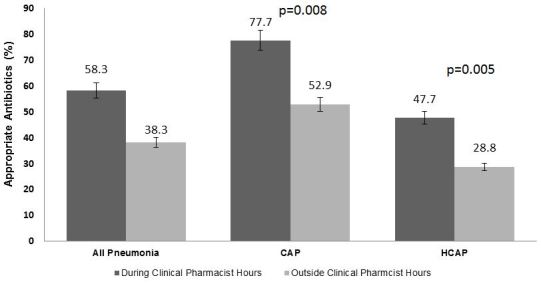
Appropriate empiric antimicrobial therapy. *CAP*, community-acquired pneumonia; *HCAP*, healthcare-associated pneumonia.

**Figure 2:**
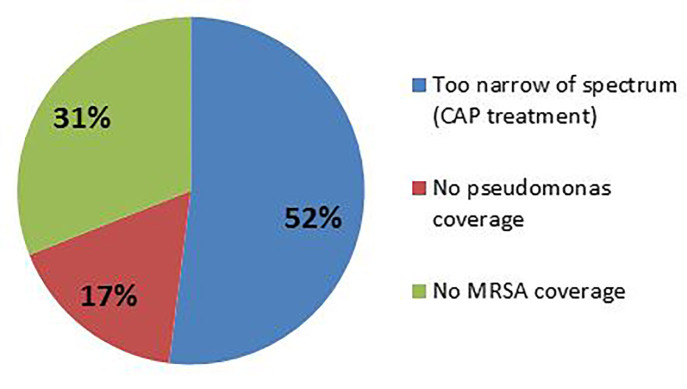
Categorization of inappropriate antimicrobial therapy description for patients presenting with HCAP (Healthcare-associated pneumonia). *CAP*, Community-acquired pneumonia.

**Figure 3:**
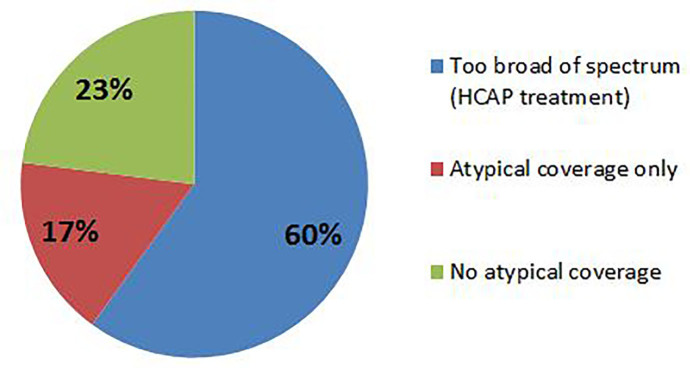
Categorization of inappropriate antimicrobial therapy description for patients presenting with CAP(community-acquired pneumonia). *HCAP*, healthcare-associated pneumonia.

Patients who received antimicrobial therapy and were seen in the ED when an EM pharmacist was present were significantly more likely to receive appropriate antimicrobial therapy (58.3% vs. 38.3%; p<0.001). Significance remained regardless of pneumonia subtype (CAP vs. HCAP) (Figure 1). The main reason for inappropriate antimicrobial therapy was misidentification of pneumonia subtype (e.g., treating using HCAP antibiotics for CAP, or vice versa) (Figure 2 and 3). There were no statistical differences in time to first antibiotic or in secondary clinical outcomes (Table 3). Using univariate analysis among survivors, 30-day repeat visits were not associated with the presence of an EM pharmacist. Using multivariable logistic regression adjusting for appropriate antimicrobial therapy and HCAP, 30-day repeat visits were more likely in patients diagnosed with HCAP (aOR 3.08 [1.49–6.35], p=0.002), but was not associated with the presence of an ED pharmacist (aOR 1.718 [0.915–3.23], p=0.09). Patients who received appropriate empiric antimicrobial therapy in the ED were significantly more likely to have the same therapy continued after admission to the hospital (65% vs. 35%, p<0.001).

## DISCUSSION

Appropriate antimicrobial therapy for pneumonia decreases hospital LOS and mortality.^9^,^17^,^18^ The ED is the primary source of admission for the majority of pneumonia patients, making it an optimal place to improve empiric antimicrobial selection.^3^,^19^ Our study differs from previous research as it evaluated the effects of the presence of an EM clinical pharmacist on appropriate antimicrobial therapy for all patients presenting with pneumonia (CAP and HCAP). The results shed light on the ability of a clinical pharmacist to impact appropriate empiric antimicrobial therapy for pneumonia in the ED.

Because appropriate antimicrobial therapy improves clinical outcomes, it is important that patients receive therapy covering presumed pathogens without exposing them to unnecessarily broad therapy resulting in increased resistance and adverse effects.^20^,^21^

Our findings are important for several reasons. First, even though guidelines exist to provide recommendations for empiric treatment of CAP and HCAP, adherence improved when a clinical pharmacist was present in the ED. In our study, over half of the patients treated when the EM clinical pharmacist was present received appropriate therapy. In contrast to previous studies evaluating pneumonia, our findings showed improvement in a higher percentage of patients receiving guideline-concordant therapy for CAP and HCAP when an EM pharmacist was present.^8^,^10^

Second, our study showed that guideline adherence was low for both CAP and HCAP when the clinical pharmacist was not present. A previous study by DeFrates et al. evaluated the presence of an EM clinical pharmacist on appropriate antimicrobial therapy in patients presenting to the ED with HCAP. 10 They were able to show that the presence of an EM clinical pharmacist improves appropriate therapy for HCAP (49.4% vs. 25.7%, p=0.005). 10 Our HCAP population findings were similar to DeFrates et al., as our treatment and control group received guideline-concordant therapy approximately 50% and 25% of the time respectively. 10 However, they did not evaluate antimicrobial therapy for patients admitted with CAP.

Third, patients who received appropriate antimicrobial therapy were significantly more likely to have that therapy continued upon admission. Previous reports have shown that care received in the ED can positively or negatively influence care after the patient is admitted to the hospital. 22–24 Our findings support a positive impact; however, interventions to improve antimicrobial selection should continue to focus on improving the percentage of patients receiving appropriate antimicrobial therapy in the ED.

Survival of pneumonia patients has been shown to be directly related with early appropriate antimicrobial therapy.^17^,^18^,^25^ Although a definitive time point has not been established to provide timely therapy, the median time to antimicrobial therapy in both groups was approximately two hours after being admitted to the ED. This time frame was significantly shorter compared to previous reports and within an acceptable time period to decrease mortality based on previous evidence.^10^,^17^ However, we were unable to show a significant difference in mortality even though a significantly higher proportion of control patients received inappropriate antimicrobial therapy. While our study was not powered to evaluate the effects of appropriate antimicrobial therapy on mortality or other clinical outcomes, this remains an important finding as exposure to inappropriate antimicrobial therapy can lead to serious adverse drug reactions and development of MDR pathogens.^20^,^21^,^26^

Our results show that patients often receive inappropriate antimicrobial therapy regardless of pneumonia type (CAP vs. HCAP). It is evident that an EM clinical pharmacist can play an important role in intervening on antimicrobial orders in the ED to promote guideline-concordant antimicrobial selection for patients presenting with pneumonia. Additionally, EM clinical pharmacists should continue to enhance their role in improving the identification of patients at risk for MDR pathogens, appropriate dosing based on patient-specific characteristics and timely administration of antimicrobial therapy for patients with pneumonia.

## LIMITATIONS

This study has several important limitations. First, because it was retrospective our study introduced the possibility of unmeasured confounders potentially influencing the outcome. However, both groups had similar baseline demographics and a similar proportion of pneumonia type (CAP and HCAP), so it is likely that internal validity of our study was maintained. Second, some of our data could be incompletely recorded, especially risk factors for HCAP. The majority of the risk factors for HCAP should be accurately recorded (e.g. dialysis dependent, residence in a long-term care facility or nursing home, immunosuppression); however, we cannot be sure that all relevant factors were consistently captured. Additionally, because we are not a closed healthcare system, repeat visits at 30 days could have been underreported if the patient presented to a different ED.

Third, patient group assignment was based on antimicrobial therapy administration times in the EMR. By assigning patients in this manner, patients could have been placed in the control group even though antimicrobial recommendations were made before the end of the clinical pharmacist’s shift. On the other hand, patients could have been assigned the treatment group even if the patient was admitted to the ED before the arrival of the clinical pharmacist. Antimicrobial therapy could have been ordered before clinical pharmacist arrival but administered shortly after the shift started, not allowing time for the clinical pharmacist to intervene on the orders. Because both the treatment and control groups would

have patients fall into these categories, we do not believe there were any major between group differences.

Fourth, the latest iteration of the IDSA guidelines for the treatment of hospital-acquired and ventilator-associated pneumonia was recently updated and the designation of HCAP has been removed. 27 The main rationale for removing this designation is the lack of evidence showing the risk factors used to define the HCAP population are associated with a higher risk of MDR pathogens. 28–30 Additional evidence suggests HCAP-designated patients receiving broad-spectrum therapy show no improvement in clinical cure rates or outcomes. 31 As evidence continues to evolve and the prevalence of resistant pathogens increases, it is imperative yet remains complex to identify patients who require broad-spectrum antimicrobial therapy.

Fifth, we elected to use *ICD-9* codes to identify pneumonia patients. Using *ICD-9* codes has been shown to be an effective approach to identify patients in our cohort; however, we could not rule out that some patients might have been missed because of improper coding. 10 Sixth, based on ED workflow and on competing priorities (e.g. other critically ill patients, trauma/cardiac arrest resuscitation), it was not feasible for the EM clinical pharmacist to review all antimicrobial therapy before administration by nursing staff. This could reflect why appropriateness was not higher in the intervention group.

Finally, we did not define appropriate antimicrobial therapy based on culture and susceptibility reports. The majority of patients diagnosed with pneumonia do not have positive blood cultures, and most sputum cultures are low yield with variable results influenced by the quality of the collection process. 6 Conducting a study using only culture-positive patients would not have been feasible. Additionally, it would have decreased the external validity of our study as antimicrobial therapy initiated in the ED is not typically based on culture data. 6

## CONCLUSION

The presence of an EM clinical pharmacist significantly increases the likelihood of appropriate empiric antimicrobial therapy for patients presenting to the ED with pneumonia. Future studies should focus on the impact of EM clinical pharmacist interventions on clinical outcomes for patients presenting to the ED with pneumonia. The studies should be consistent and reproducible with their findings of specific interventions to demonstrate the benefit of an EM clinical pharmacist to impact patients’ medication-related outcomes.

## Figures and Tables

**Table 1:** Baseline demographics in study examining whether the presence of a clinical pharmacist in the emergency department affects the appropriateness of antimicrobial therapy in patients presenting with pneumonia.

	Emergency medicine clinical pharmacist present	
	
	Yes (n=103)	No (n=303)
		
Age, years (mean SD)	60 (18.4)	62.4 (17.4)
Weight, kg (mean SD)	85.3 (23.5)	85.7 (27.1)
Temperature, C° (mean SD)	37.3 (1)	37.6 (1.2)
White blood cell, k/mm^3^ (mean SD)	13.2 (7)	12.7 (7.3)
Lactate, mEq/L (mean SD)	1.7 (1.1)	1.9 (1.4)
Mechanical ventilation (%)	7 (7)	14 (5)
ICU admission (%)	21 (20)	54 (18)
Community-acquired pneumonia (%)	36 (35)	119 (39)
Healthcare-associated pneumonia (%)	67 (66)	184 (61)

*ICU*, intensive care unit; *SD*, standard deviation.

**Table 2:** Healthcare-associated pneumonia risk factors.#

Risk factors	n (%)
Hospitalization for 2 days or more in the preceding 90 days	131 (52)
Residence in long term facility or nursing home	70 (28)
Chronic hemodialysis	19 (8)
Home infusion therapy	1 (<1)
Chronic home wound care	40 (16)
Immunocompromised	112 (45)

# Multiple risk factors may have been recorded for each patient.

**Table 3:** Comparison of secondary outcomes and clinical outcomes in pneumonia patients based on whether an emergency medicine clinical pharmacist was on duty.

	Clinical pharmacist coverage (n=103)	No clinical pharmacist coverage (n=303)	p value
Time to first antibiotic, hrs (median, IQR)	2.01 (1.25,2.83)	2.12 (1.35,3.48)	0.15
Average vancomycin dose, mg/kg (mean, SD)	16.7 (2.8)	17.3 (4.4)	0.32
Correct vancomycin dose, n (%)^1^	31 (81.6)	62 (71.3)	0.22
Hospital LOS (days, IQR)	4.1 (2.2,7.8)	3.9 (2.6,6.8)	0.57
In-hospital mortality, n (%)	2 (2)	11 (4)	0.40
30-day repeat hospital visits, n (%)^2^	20 (19.8)	33 (11.3)	0.03

*LOS,* length of stay; *IQR*, interquartile range; *SD,* standard deviation.

1 Analysis only among those receiving vancomycin (N=125), of which 38 were seen when the pharmacist was present and 87 when there was no pharmacist).

2 Among those who survived initial visit (N=393).
